# Enhanced Cytotoxic Effects of Combined Valproic Acid and the Aurora Kinase Inhibitor VE465 on Gynecologic Cancer Cells

**DOI:** 10.3389/fonc.2013.00058

**Published:** 2013-03-20

**Authors:** Yanfang Li, Tao Liu, Cristina Ivan, Jie Huang, De-Yu Shen, John J. Kavanagh, Robert C. Bast, Siqing Fu, Wei Hu, Anil K. Sood

**Affiliations:** ^1^Departments of Gynecologic Oncology and Reproductive Medicine, The University of Texas MD Anderson Cancer Center Houston, TX, USA; ^2^Center for RNAi and Non-Coding RNA, The University of Texas MD Anderson Cancer Center Houston, TX, USA; ^3^Departments of Experimental Therapeutics, The University of Texas MD Anderson Cancer Center Houston, TX, USA; ^4^Departments of Investigative Cancer Therapeutics, The University of Texas MD Anderson Cancer Center Houston, TX, USA; ^5^Departments of Cancer Biology, The University of Texas MD Anderson Cancer Center Houston, TX, USA

**Keywords:** valproic acid, Aurora kinase inhibitor, ovarian cancer, cervical cancer, endometrial cancer

## Abstract

Increasing evidence shows that targeting epigenetic changes including acetylation and deacetylation of core nucleosomal histones as well as Aurora kinases hold promise for improving the treatment of human cancers including ovarian cancer. We investigated whether the histone deacetylase (HDAC) inhibitor, valproic acid (VPA), and the Aurora kinase inhibitor VE465 can have additive or synergistic effects on gynecologic cancer cells. We tested the *in vitro* antitumor activity of VPA and VE465, alone and in combination, in gynecologic cancer cells and assessed potential mechanisms of action. 3-[4,5-dimethyl-2-thiazolyl]-2,5-diphenyl-2H-tetrazolium bromide (MTT) analysis revealed that 72 h of treatment with VPA or VE465 alone induced dose-dependent cytotoxic effects in nine gynecologic cancer cell lines (ovarian: 2008/C13, OVCAR3, SKOV3, and A2780; cervical: ME180 and CaSki; endometrial: HEC-1B; and uterine sarcoma: MES-SA and MES-SA/D×5). Co-treatment with VPA and VE465 enhanced cytotoxic effects on five of these cell lines: ovarian: 2008/C13, A2780, and OVCAR3; endometrial: HEC-1B; and cervical: ME180. In ovarian 2008/C13 cells, co-treatment with VPA (2 mM) and VE465 (1 μM) induced more apoptosis than either VPA or VE465 alone. Western blot analysis showed that VPA alone increased the expression of cleaved PARP and p21 in a dose-dependent manner in 2008/C13 cells, while co-treatment with VPA and VE465 induced more cleaved PARP than treatment with VPA or VE465 alone did. The combined use of VPA and VE465 enhanced cytotoxic effects in some ovarian cancer cells, via enhanced induction of apoptosis. Targeting epigenetics with the HDAC inhibitor, in combination with Aurora kinase inhibitors, holds promise for more effective therapy of ovarian cancer.

## Introduction

Gynecologic cancers remain a significant cause of mortality among American women (Jemal et al., 2007). Endometrial cancers are the most prevalent, followed by cancers of the ovaries and the uterine cervix. Approximately 25% of endometrial cancers (Hickerson, 2003), 24% of cervical cancers (Benedet et al., 2001), and 70% of ovarian cancers (O’Malley et al., 2003) are diagnosed at an advanced stage. The prognosis for patients with these advanced cancers is poor; thus, novel therapeutic strategies are urgently needed. One strategy we investigated was the potential of new drugs for targeting epigenetic alteration, specifically histone deacetylase (HDAC) inhibitors, which are emerging as a new class of potential anticancer agents.

Acetylation and deacetylation of core nucleosomal histones play important roles in the epigenetic regulation of gene expression (Marks et al., 2000). At least two classes of enzymes are involved in controlling the acetylation of histones: histone acetyltransferases (HATs) and HDACs. HDACs catalyze the removal of acetyl groups on the amino-terminal lysine residues of histones, which generally results in transcriptional repression (Grunstein, 1997; Mai et al., 2005) and aberrant silencing of tumor-suppressor genes (Marks et al., 2000). Increasing evidence shows that aberrations in HAT or HDAC activity and in histone acetylation are linked to the development of certain cancers (Cress and Seto, 2000; Pandolfi, 2001; Timmermann et al., 2001; Verdin et al., 2003), including gynecologic cancers (Caslini et al., 2006; Hrzenjak et al., 2006). Inhibition of HDACs increases histone acetylation and may lead to the restoration of transcriptionally silenced pathways or the suppression of aberrantly expressed genes (Richon and O’Brien, 2002). Increasing numbers of HDAC inhibitors are being investigated, and several (e.g., phenylbutyrate and suberoylanilide hydroxamic acid) are being tested in clinical trials (Balch et al., 2004).

Valproic acid (VPA) is a short-chain fatty acid that has been used to treat epilepsy for 30 years. Recently, VPA has been shown to inhibit proliferation in various cancers, including gynecologic cancers [e.g., endometrial (Takai et al., 2004a), ovarian (Takai et al., 2004b), and cervical cancers (de la Cruz-Hernandez et al., 2007) and uterine sarcoma both *in vivo* and *in vitro* (Hrzenjak et al., 2006)]. Although many mechanisms of action may underlie the antitumor activity of VPA, many studies have suggested that modulating the epigenome by inhibiting HDACs is one of the main actions of VPA (Gottlicher et al., 2001; Phiel et al., 2001; Blaheta et al., 2005). VPA promotes differentiation by inhibiting HDACs, which in turn results in the re-expression of epigenetically mediated inactivated genes that are involved in cellular differentiation and development (Gurvich et al., 2004); cell cycle arrest at the G_1_/S boundary mediated by the Rb and related proteins associated with the p53-independent induction of p21^WAF1/CIP1^ and the repression of cyclins; the activation of the G_2_/M phase by initiating a G_2_-phase checkpoint; and apoptosis via the death-receptor and mitochondrial death pathways (Facchetti et al., 2004).

Valproic acid is highly effective in suppressing the growth of human ovarian carcinoma cells (Takai et al., 2004b). Clonogenic assays have shown that all ovarian carcinoma cell lines are sensitive to the growth-inhibitory effects of VPA. The prominent arrest of malignant cells in the G_0_/G_1_ phase of the cell cycle is likely to account for this effect by the increased expression of p21^WAF1^ and p27^KIP1^, accompanied by the accumulation of acetylated histones H3 and H4 (Takai et al., 2004b).

Targeting Aurora kinases is another potential therapeutic strategy in cancer treatment (Fu et al., 2006). Three human Aurora kinases (A, B, and C) have been cloned (Fu et al., 2006) and mapped to chromosomes 20q13.2, 17p13.1, and 19q13.43, respectively (Li et al., 2004; Wheatley et al., 2004). Aurora kinases play a crucial role in controlling chromosome movement and organization during mitosis. Aurora kinase A, a serine-threonine protein kinase, is essential for mitotic spindle formation and accurate chromosome segregation (Adams et al., 2001). Aurora kinase B, a chromosome passenger protein kinase, contributes to centrosome separation, chromosome segregation, and cytokinesis (Adams et al., 2001). Aurora kinase C, normally found only in germ cells, is also a chromosome passenger protein kinase, and is able to complement the loss of Aurora kinase B expression under some circumstances (Li et al., 2004; Sasai et al., 2004).

Increasing evidence shows that Aurora kinases are involved in tumorigenesis (Fu et al., 2006). They are frequently overexpressed and amplified in human cancers (Zhou et al., 1998), including ovarian (Gritsko et al., 2003; Hu et al., 2005) and endometrial cancers (Moreno-Bueno et al., 2003), and are therefore potential targets for anticancer therapy (Naruganahalli et al., 2006; Yang et al., 2006). A number of Aurora kinase inhibitors (e.g., VE465, VX-680, and AT-9283) have been developed (Naruganahalli et al., 2006), and their anticancer efficacy has been shown in preclinical studies and phase 1 and 2 trials (Carvajal et al., 2006; Naruganahalli et al., 2006). Treatment with these potent compounds has resulted in the arrest of proliferation in various tumor cell lines, including the human ovarian cancer cell line A2780, and in the inhibition of phosphorylation of histone H3 on serine 10 (Fancelli et al., 2005, 2006). It is therefore likely that targeting the enzymes involved in controlling histone modification by processes such as acetylation and phosphorylation will provide new, better therapeutic opportunities for ovarian cancer. However, studies are needed to determine the cytotoxic action of Aurora kinase inhibitors against gynecologic cancers and whether VE465 can sensitize gynecologic cancers to other antitumor drugs. In this study, we investigated whether VE465 can enhance the cytotoxic effect of VPA on gynecologic cancer cells and the possible mechanisms of action involved.

## Materials and Methods

### Reagents and cell culture

We dissolved VPA (Sigma-Aldrich, St. Louis, MO, USA) in distilled water at a stock concentration of 1 M and filtered through a 0.2-μm filter. We dissolved VE465 (Vertex Pharmaceuticals, Cambridge, MA, USA) in dimethyl sulfoxide at a stock concentration of 10^−2^ M. Experiments were conducted on nine established gynecologic cancer cell lines, including four ovarian (three platinum-resistant: 2008/C13, OVCAR3, and SKOV3, and one platinum-sensitive: A2780), two cervical (paclitaxel-resistant ME180 and cisplatin-resistant CaSki; Saxena et al., 2005), one endometrial (carboplatin-resistant HEC-1B; Smith et al., 2004), and two uterine sarcoma (multiple-drug-resistant MES-SA and its subline MES-SA/D×5) cell lines. The ovarian cancer cell lines were obtained from Dr. Ralph S. Freedman (The University of Texas MD Anderson Cancer Center, Houston, TX, USA) (Melichar et al., 2007). The cervical and endometrial cancer cell lines and uterine sarcoma cell lines were purchased from the American Type Culture Collection (Manassas, VA, USA).

The ovarian cancer and uterine sarcoma cells were cultured in RPMI 1640 medium (Gibco BRL, Grand Island, NY, USA) supplemented with 10% fetal calf serum. The cervical and endometrial cancer cells were propagated in a medium recommended by the American Type Culture Collection. For drug treatment, cells were grown to about 90% fluency and detached by 0.5% ethylenediaminetetraacetic acid (EDTA)-trypsin (Gibco BRL).

### MTT assay

We determined cell growth and viability using the MTT method with an EZ4U kit (American Laboratory Products Company, Windham, NH, USA). This assay, based on the transformation of tetrazolium salt into colored soluble formazans as a result of the mitochondrial activity of the viable cells, determines the percentage of viable cells. Cells were seeded in a 96-well plate at 4000 cells per 200 μL of medium per well, and were allowed to adhere to the plate overnight. The next day, the cells were treated with VPA and VE465, alone and in combination, at indicated concentrations. After treatment for 72 h, we performed the MTT assay according to the manufacturer’s instructions. We measured spectrophotometric absorbance of each sample at 490 nm using a microplate reader (BioTek Instruments, Winooski, VT, USA), performing this four times for each drug concentration in each experiment. The percentage of cell survival was determined by the ratio of absorbance of the sample to that of the control. The combination effect of VE465 and VPA was evaluated by the method described by Chou and Talalay (Chou, 2006, 2010) using R (version 2.14.2) and a combination index (CI) was calculated. A CI < 1 indicates a synergistic interaction, CI = 1 is additive, and CI > 1 is antagonistic (Marth et al., 1986; Hu et al., 2002).

### Flow cytometry

We seeded 1.8 × 10^6^ cells in T25 flasks (25 m^2^) and allowed them to adhere to the flask overnight. The next day, the cells were treated with VPA and VE465, alone and in combination. After treatment for 72 h, the cells were harvested using 0.5% EDTA-trypsin, washed three times with ice-cold 1 × phosphate-buffered saline (PBS), and fixed with 70% ethanol at room temperature for 15 min. Cell pellets were then stained with 50 μg/mL of propidium iodide (Calbiochem, San Diego, CA, USA) and 20 μg/mL of ribonuclease A (Sigma-Aldrich, St. Louis, MO, USA) at room temperature for 15 min. The fraction of cells that were in the sub-G_1_ phase was determined by using a flow cytometer (Epics XL-MCL, Beckman Coulter, Miami, FL, USA).

### TUNEL assay

Cells were harvested with 0.5% EDTA-trypsin and washed with ice-cold 1 × PBS. Cytospin-prepared slides were fixed with 4% paraformaldehyde for 20 min at room temperature, washed with PBS, air dried, and then stored at −20°C until use. A terminal deoxynucleotidyl transferase-mediated dUTP nick-end labeling (TUNEL) assay was performed by using an Apo-BRDU-IHC kit (catalog no. AH1001, Chemicon International, Temecula, CA, USA) according to the manufacturer’s instructions.

### Western blot analysis

For protein extraction, cells were cultured in T25 flasks and treated as described above. After the cells were harvested, they were washed three times with ice-cold 1 × PBS and lysed with modified radioimmunoprecipitation lysis buffer [10 mM Tris HCl (pH 8.0), 10 mM EDTA (pH 8.0), 0.15 M NaCl, 1% NP40, and 0.5% sodium dodecyl sulfate] containing freshly added protease inhibitor. After 30 min of incubation on ice, we cleared the lysates by centrifugation at 12,000 rpm and 4°C for 30 min.

We quantified protein concentrations by using the Bio-Rad protein assay (Bio-Rad Laboratories, Hercules, CA, USA). Thirty-five micrograms of protein were separated with 8–15% sodium dodecyl sulfate-polyacrylamide-gradient gel (Bio-Rad Laboratories), transferred to a nitrocellulose membrane (Amersham Biosciences, Buckinghamshire, UK), and probed with the following diluted antibodies: poly (ADP-ribose) polymerase (PARP; 1:800; Promega Corporation, Madison, WI, USA), glyceraldehyde-3-phosphate dehydrogenase, used as an internal control; 1:2,500; Santa Cruz Biotechnology, Santa Cruz, CA, USA), and p21 (cyclin-dependent kinase-interacting protein or WAF1/Cip1; 1:200, Santa Cruz Biotechnology). Signals were visualized on reaction with an enhanced chemiluminescence detection reagent (Amersham Biosciences, Piscataway, NJ, USA).

### Statistical analysis

We calculated the concentrations of VPA and VE465 that inhibited 50% of the cells (IC_50_) using SigmaPlot 8.0 software (Systat Software, San Jose, CA, USA). All numerical data were expressed as mean ± standard deviation. We determined the significance of the difference between the two groups with an independent-samples *t*-test. A *P*-value of <0.05 was considered statistically significant.

## Results

### Growth inhibition by VPA or VE465 alone in gynecologic cancer cells

**Figure 1:**
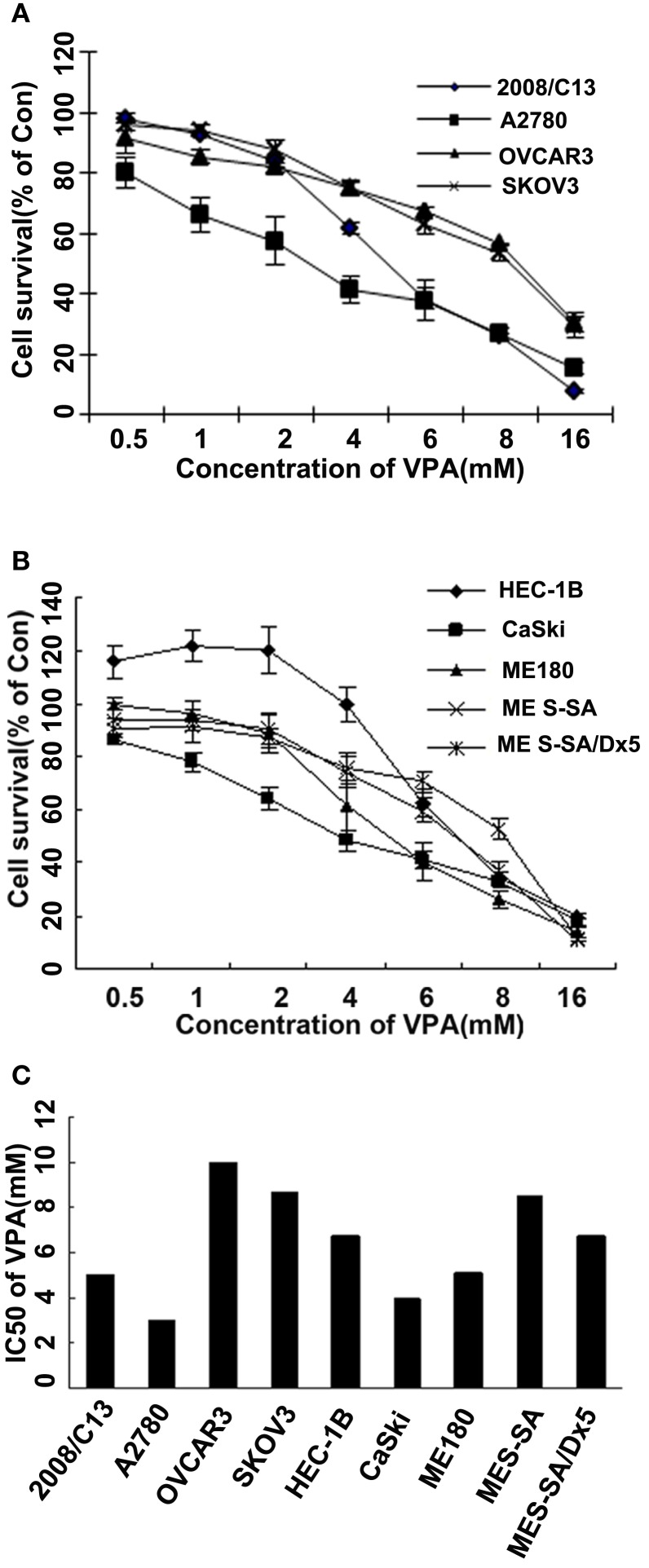
**Cytotoxicity in gynecologic cancer cell lines after 72 h of treatment with VPA alone**. **(A)** Growth curves for ovarian cancer cell lines (2008/C13, A2780, OVCAR3, and SKOV3) after treatment with VPA alone at increasing concentrations (0.5–16 mM). **(B)** Growth curves for uterine and cervical cancer cell lines (ME180, CaSki, HEC-1B, MES-SA, and MES-SA/D×5) after treatment with VPA alone at increasing concentrations (0.5–16 mM). **(C)** IC_50_ of VPA treatment in the nine gynecologic cancer cell lines studied.

**Figure 2:**
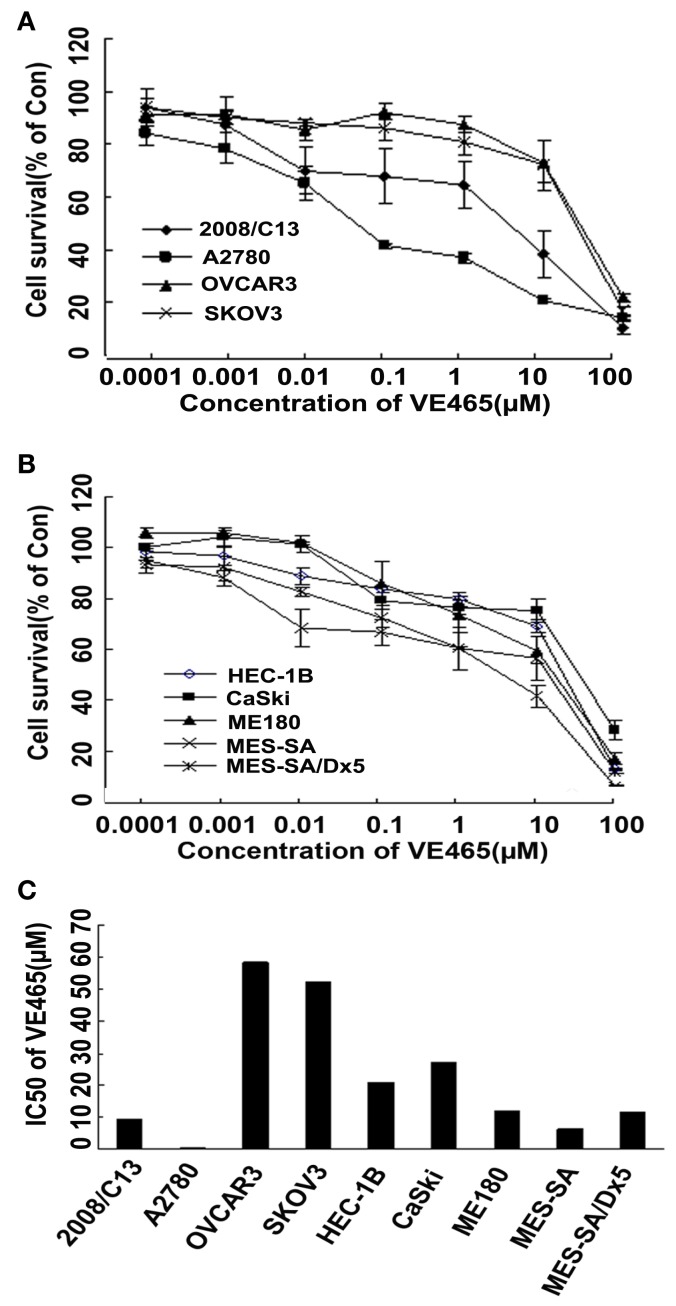
**Cytotoxicity in gynecologic cancer cell lines after 72 h of treatment with VE465 alone**. **(A)** Growth curves for ovarian cancer cell lines (2008/C13, A2780, OVCAR3, and SKOV3) after treatment with VE465 alone at increasing concentrations (0.0001–100 μM). **(B)** Growth curves for uterine and cervical cancer cell lines (ME180, CaSki, HEC-1B, MES-SA, and MES-SA/D×5) after treatment with VE465 alone at increasing concentrations (0.0001–100 μM). **(C)** IC_50_ of VE465 in various gynecologic cancer cell lines.

We tested the cytotoxic effect of VPA (0.5–16.0 μM) and VE465 (0.0001–100 μM) as single agent. After 72 h of treatment, cell survival was measured with an MTT assay. As shown in Figures 1 and 2, both VPA and VE465 induced dose-dependent cytotoxic effects in the gynecologic cancer cell lines. The IC_50_ values for VPA were between 3.0 and 10.0 mM; the values for VE465 were between 0.1 and 58.3 μM (Table 1).

**Table 1:** **IC_50_ of VPA and VE465 after 72 h of treatment in various cell lines**.

Cell line	VPA (mM)	VE465 (μM)
2008/C13	5.0	9.3
A2780	3.0	0.1
OVCAR3	10.0	58.3
SKOV3	8.7	52.3
HEC-1B	6.7	20.9
CaSki	4.0	27.4
ME180	5.1	12.2
MES-SA	8.5	6.4
MES-SA/D×5	6.7	11.5

*VPA, valproic acid*.

### Enhanced cytotoxic effect of combined VPA and VE465 on gynecologic cancer cells

To evaluate the combined cytotoxic effects of VPA and VE465 on the nine gynecologic cancer cell lines, we performed a 72-h co-treatment using both agents. We used VPA at a series of concentrations ranging from 0.5 to 16.0 mM and VE465 at concentrations of 0.1, 1, and 10 μM for all cell lines but A2780. For the A2780 cell line, we used three lower levels of the VE465 concentration (0.01, 0.1, and 1 μM) because of the cells’ high degree of sensitivity to VE465.

**Figure 3:**
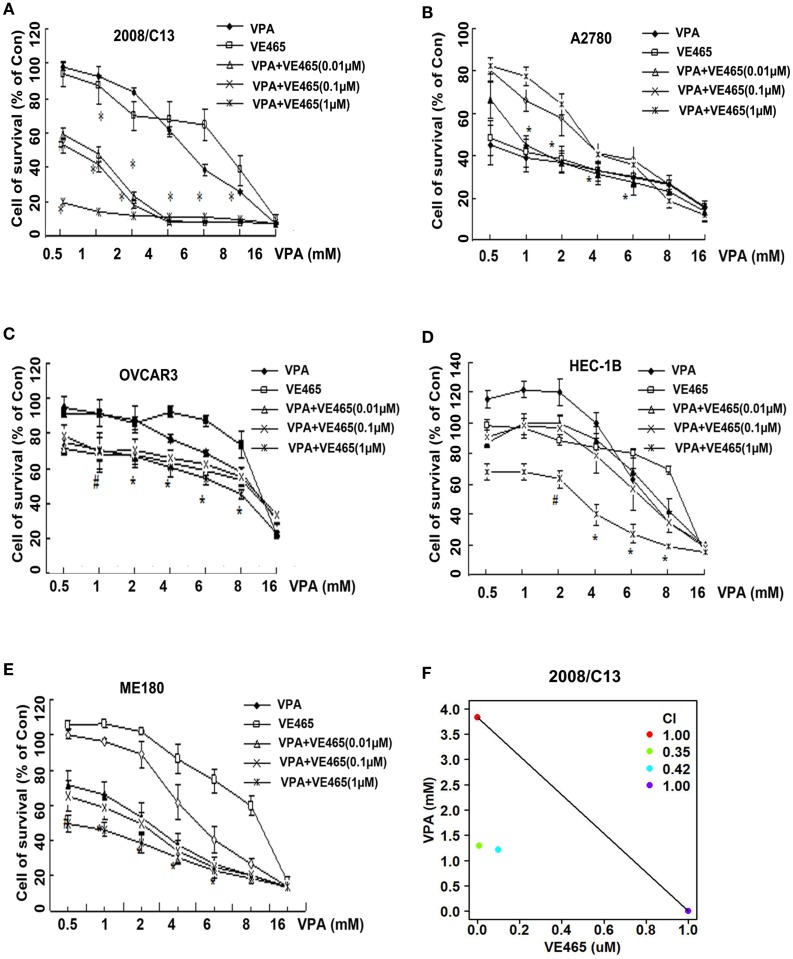
**Cytotoxicity in gynecologic cancer cell lines: (A) 2008/C13, (B) A2780, (C) OVCAR3, (D) HEC-1B, and (E) ME180 – after 72 h of co-treatment with VPA and VE465 at increasing concentrations**. **(F)** Isobologram analysis of the combined effects of VE465 and VPA in 2008C13 cells. A combination index (CI) < 1 indicates a synergistic interaction, CI = 1 is additive, and CI > 1 is antagonistic.

We observed a synergistic effect of VPA and VE465 on 2008/C13 ovarian cancer cells. In cell line 2008/C13, IC_50_ decreased from 5.0 mM for VPA used alone to ≤1 mM for VPA combined with various concentrations of VE465 (Table 2). When the concentration of VPA was between 2 and 8 mM, adding 0.1, 1, or 10 μM VE465 induced substantial cell growth inhibition (76.7–92.7%), which was significantly greater than that induced by VPA or VE465 alone (all *P* < 0.001). When the concentration of VPA was reduced to <2 mM and 10 μM VE465 was added, cell growth inhibition was 80.3–85.7%; when 0.1 or 1 μM VE465 was added, inhibition was 40.5–58.4%. At these concentration ranges, the growth inhibition induced by combined treatment was significantly greater than that induced by VPA or VE465 alone (*P* < 0.05) (Figure 3A). Two drug combination analysis with Chou and Talalay method indicates that a synergistic interaction occurred between VPA and VE465 (CI < 1; Figure 3F).

**Table 2:** **Decreased IC_50_ of VPA (mM) in combined treatment with VE465 in gynecologic cancer cells**.

Treatment	VPA IC_50_				
	A2780 cells	2008/C13 cells	OVCAR3 cells	HEC-1B cells	ME180 cells
Single agent VPA	3.0	5.0	10.0	6.7	5.1
VPA + VE465 (0.01 μM)	1.0	NA	NA	NA	NA
VPA + VE465 (0.1 μM)	<0.5*	0.9	9.0	7.3	2.4
VPA + VE465 (1 μM)	<0.5*	0.6	9.8	6.5	1.9
VPA + VE465 (10 μM)	NA	<0.5*	6.6	3.2	<0.5*

*VPA, valproic acid; NA, not available*.

*Even though the concentration of VPA used in combination with VE465 was as low as 0.5 mM, the growth inhibition was >50%, so the exact IC_50_ could not be obtained by the program.

With the A2780 cells, growth was inhibited 55.8–72.4% when the concentration of VPA was 1–6 mM and the concentration of VE465 was 0.01 μM, which was significantly greater than the growth inhibition induced by VPA or VE465 alone (all *P* < 0.001) (Figure 3B). The IC_50_ decreased from 3.0 mM with VPA used alone to ≤1 mM when VPA was combined with various concentrations of VE465 (Table 2).

Similarly, greater growth inhibition was induced by combined treatment of VPA and VE465 than by VPA or VE465 alone in OVCAR3 (for 1–6 mM VPA and 0.1, 1, and 10 μM VE465) (Figure 3C), HEC-1B (for 2–8 mM VPA and 10 μM VE465) (Figure 3D), and ME180 cells (for 0.5–8 mM VPA and 0.1, 1, and 10 μM VE465) (Figure 3E). The IC_50_ of VPA was decreased when VE465 was added (Table 2).

### Apoptosis induced by VPA and VE465, alone and in combination, in 2008/C13 cells

**Figure 4:**
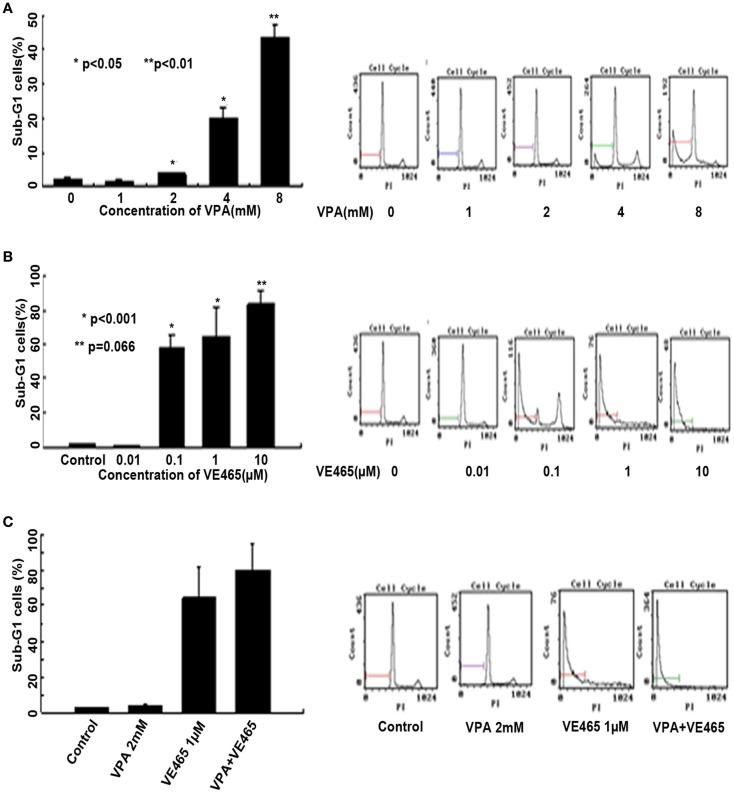
**Flow cytometric analysis of 2008/C13 cells after 72 h of treatment with VPA or VE465, alone and in combination**. **(A)** Treatment with VPA alone with increasing concentrations (1–8 mM). **(B)** Treatment with VE465 alone at increasing concentrations (0.01–10 μM). **(C)** Co-treatment with VPA (2 mM) and VE465 (1 μM).

We examined apoptosis induced by VPA and VE465, alone and in combination, in the 2008/C13 cell line by using flow cytometry, TUNEL, and Western blot analysis. First, flow cytometry using propidium iodide staining showed that the percentage of sub-G_1_ cells induced by VPA at 2, 4, and 8 mM was significantly higher than that in the control (*P* < 0.05 or <0.01) (Figure 4A). A 72-h treatment of 1–8 mM VPA alone or 0.01–10 μM VE465 alone induced sub-G_1_ cells in 2008/C13 in a dose-dependent manner (Figures 4A,B). The percentage of sub-G_1_ cells induced by VE465 at 0.1 and 1 μM was also significantly higher than that in the control (*P* ≤ 0.001) (Figure 4B). The percentage of sub-G_1_ cells elicited by combined treatment of the two drugs (2 mM VPA and 1 μM VE465) was 80.1%, which was 12% higher than that induced by VE465 (64.2%) or VPA (3.7%) alone, respectively (Figure 4C).

**Figure 5:**
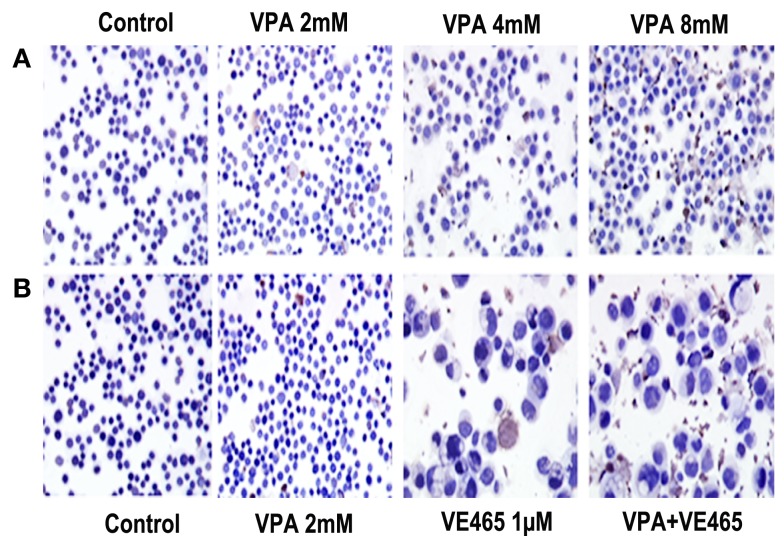
**TUNEL analysis of apoptosis induced by 72 h of treatment with VPA alone and in combination with VE465 in 2008/C13 cells**. **(A)** Treatment with VPA alone at increasing concentrations of 2–8 mM. **(B)** Co-treatment with VPA (2 mM) and VE465 (1 µM). The experiments presented in **(A,B)** were carried out at the same time and the control presented in both panels was the same.

**Figure 6:**
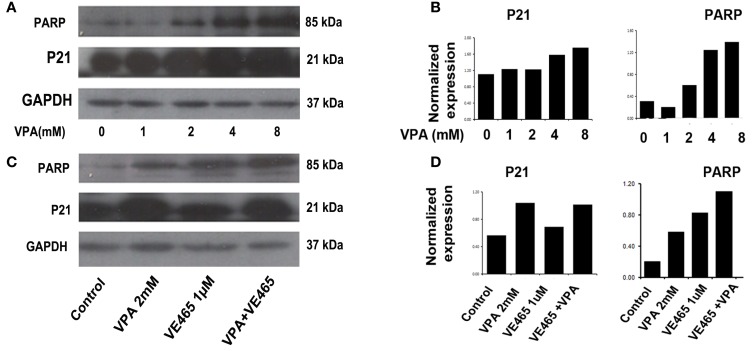
**Western blot and image analysis of cleaved PARP and P21 expression induced by 72 h of treatment with VPA alone and in combination with VE465 in 2008/C13 cells**. **(A,B)** Treatment of VPA alone induced cleaved PARP and P21 expression in a dose-dependent manner. **(C,D)** Co-treatment with VPA and VE465 induced more expression of cleaved PARP, but not of P21, than treatment with VPA or VE465 alone.

In addition, TUNEL assays showed that VPA alone increased apoptosis in cells with increasing concentration (Figure 5A) and that co-treatment with VPA (2 mM) and VE465 (1 μM) induced more apoptosis than VPA or VE465 alone did (Figure 5B). Furthermore, Western blot analysis showed that VPA induced the cleaved PARP in a dose-dependent manner and that co-treatment with VPA and VE465 induced more PARP than VPA or VE465 alone (Figures 6A,B).

### p21 expression induced by VPA and VE465, alone and in combination, in 2008/C13 cells

Since it has been suggested that HDAC inhibitors induce increased expression of p21 in various tumor cells including ovarian cancer (Richon and O’Brien, 2002; Takai et al., 2004a; Garcia-Manero et al., 2006; Hrzenjak et al., 2006), we therefore examined whether p21 expression could be affected by 72 h of treatment with VPA and VE465, alone and in combination, in 2008/C13 cells. Treatment with VPA alone induced p21 expression in a dose-dependent manner at increasing concentrations (1–8 mM). However, co-treatment with VPA and VE465 did not induce more p21 expression than VPA or VE465 alone did (Figures 6C,D).

## Discussion

The key finding of our study is that co-treatment with VPA and VE465 had enhanced antitumor effects in chemotherapy-resistant gynecologic cancer cells. One possible mechanism of action is enhanced induction of apoptosis. Growing evidence suggests that HDAC inhibitors (such as VPA) and Aurora kinase inhibitors (such as VE465 and VX-680) are effective agents against gynecologic cancers. Targeting epigenetic targets with HDAC inhibitors may enhance the antitumor activities of Aurora kinases inhibitors in ovarian cancer cells. We showed here the synergistic effect of treatment with VPA and VE465 on ovarian cancer cells (2008C13), via enhanced induction of apoptosis. The precise mechanism(s) underlying these synergistic effects require additional work.

The antitumor effect of the Aurora kinase inhibitor MK-0457 (previously called VX-680) against gynecologic cancer was reported in our previous study (Lin et al., 2008). MK-0457 alone significantly reduced tumor growth and treatment of MK-0457 and docetaxel produced even greater benefit (Lin et al., 2008). We observed that both VPA and VE465, when used alone, induced apoptosis in a dose-dependent manner. Furthermore, we found that co-treatment with VPA and VE465 significantly induced apoptosis (by flow cytometry and TUNEL assay) and more cleavage of PARP (by Western blot analysis) than did VPA or VE465 alone.

p21 is a cyclin-dependent kinase inhibitor that binds to cyclin-dependent kinase complexes, decreases kinase activity, and may play an important role in G_0_–G_1_ accumulation (Li et al., 2005). Studies have shown that HDAC inhibitors induce increased expression of p21 in various tumor cells (Richon and O’Brien, 2002; Takai et al., 2004a; Garcia-Manero et al., 2006; Hrzenjak et al., 2006), which then causes cell cycle arrest. In ovarian cancer, p21 was induced by suberoylanilide hydroxamic acid, another HDAC inhibitor (Takai et al., 2004b). We found that VPA increases p21 expression in a dose-dependent manner, suggesting that p21 is involved in the antitumor activity of VPA in ovarian cancer cells. However, we observed no significant increase in p21 expression in the cells treated with the combination of VE465 and VPA. This is inconsistent with another study in which the increased expression of p21^WAF1^ and p27^KIP1^, accompanied by the accumulation of acetylated histones H3 and H4, appeared to induce the prominent arrest of malignant cells in the G_0_/G_1_ phase of the cell cycle (Takai et al., 2004b).

Moreover, our previous study indicated that VE465 synergistically enhanced cytotoxic effects of carboplatin in ovarian cancer cells through induction of apoptosis and downregulation of phosphorylated histone 3. Whether the synergistic or parallel pathways of histone 3 phosphorylation and acetylation played a critical role in the induction of genes was not clear, however. It is plausible to hypothesize that this synergy might be explained by several causes, such as upregulating genes involved in the cell cycle (p21 and p27), the stabilization of p53, and the induction of apoptosis and balances between histone 3 phosphorylation and acetylation in controlling gene transcriptional activation. The effect of the interaction of H3 acetylation and phosphorylation on p21 expression in ovarian cancer cells treated with this combination needs to be further investigated.

In summary, combined VPA and VE465 enhanced cytotoxic effect on some gynecologic cancer cells. The possible mechanisms may be achieved via induction of apoptosis. Further studies are warranted to investigate the *in vivo* antitumor effect of these two drugs in gynecologic cancers. Synergistic activity in cell culture could translate into substantial clinical antitumor activity with the levels of each drug that can be attained clinically.

## Conflict of Interest Statement

The authors declare that the research was conducted in the absence of any commercial or financial relationships that could be construed as a potential conflict of interest.
