# Corrigendum: Enhanced Cytotoxic Effects of Combined Valproic Acid and the Aurora Kinase Inhibitor VE465 on Gynecologic Cancer Cells

**DOI:** 10.3389/fonc.2018.00009

**Published:** 2018-02-09

**Authors:** Yanfang Li, Tao Liu, Cristina Ivan, Jie Huang, De-Yu Shen, John J. Kavanagh, Robert C. Bast, Siqing Fu, Wei Hu, Anil K. Sood

**Affiliations:** ^1^Department of Gynecologic Oncology and Reproductive Medicine, The University of Texas MD Anderson Cancer Center, Houston, TX, United States; ^2^Center for RNAi and Non-Coding RNA, The University of Texas MD Anderson Cancer Center, Houston, TX, United States; ^3^Department of Experimental Therapeutics, The University of Texas MD Anderson Cancer Center, Houston, TX, United States; ^4^Department of Investigative Cancer Therapeutics, The University of Texas MD Anderson Cancer Center, Houston, TX, United States; ^5^Department of Cancer Biology, The University of Texas MD Anderson Cancer Center, Houston, TX, United States

**Keywords:** valproic acid, aurora kinase inhibitor, ovarian cancer, cervical cancer, endometrial cancer

In the original article, while there was no error in the legend for Figures 5A,B as published, we would like to clarify the apparent duplication of the control in the Figures 5A,B, which was because the experiments presented in the Figures 5A,B were carried out at the same time and the control presented in both panels was the same. The edited legend appears below. The authors would like to state that this does not change the scientific conclusions of the article in any way. Additional supplementary figures are also provided below to corroborate our originally published scientific conclusions.

**Figure 5** TUNEL analysis of apoptosis induced by 72 h of treatment with VPA alone and in combination with VE465 in 2008/C13 cells. **(A)** Treatment with VPA alone at increasing concentrations of 2–8 mM. **(B)** Co-treatment with VPA (2 mM) and VE465 (1 µM). The experiments presented in **(A,B)** were carried out at the same time and the control presented in both panels was the same.

The original article was updated.

## Supplementary Documents

**Figure S1:**
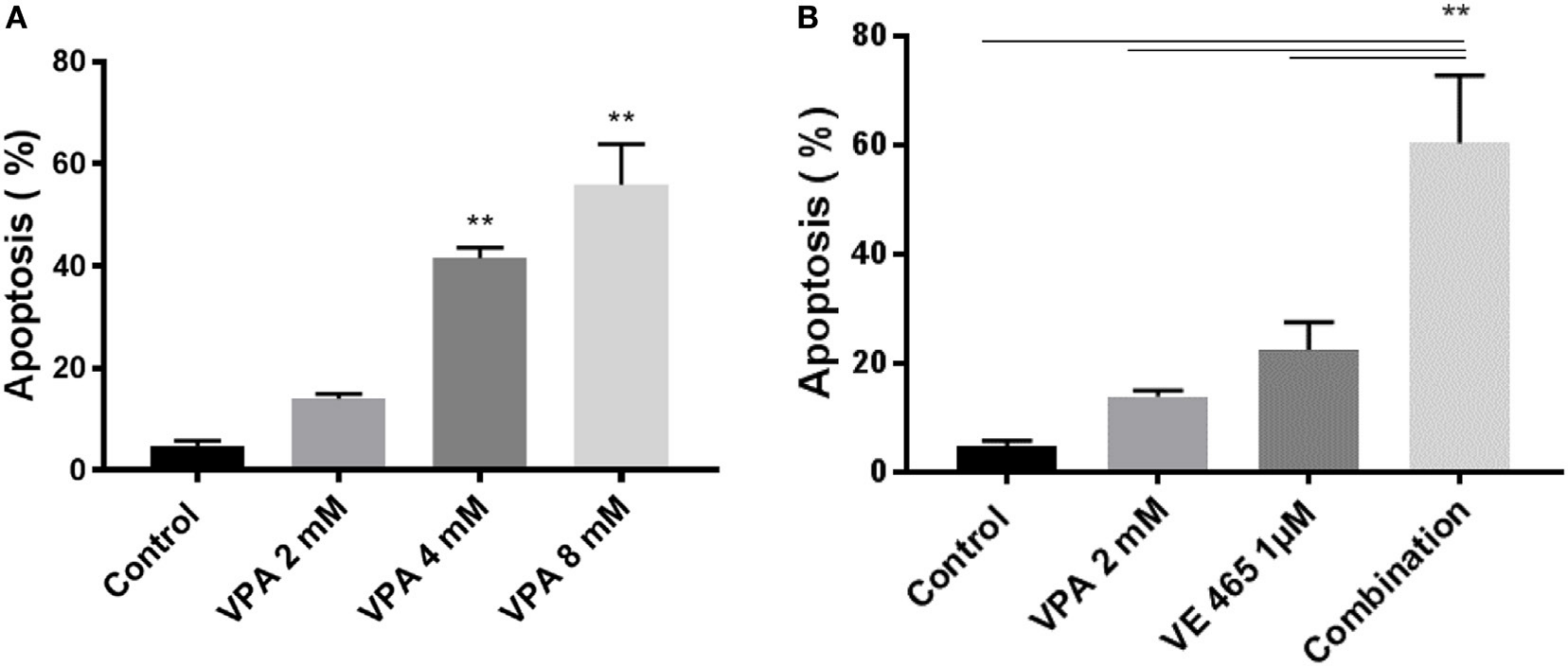
(relates to Figure 5). Annexin V analysis of apoptosis induced by VPA alone or in combination with VE 680 (VE465) for 72 h in 2008/C13 cells. Apoptotic cells were evaluated using an Annexin V apoptosis detection kit (BD Biosciences) according to the manufacturer’s protocol. Briefly, 2008/C13 cells (3 × 10^5^ per well) were plated on 6-well plates overnight. The cells were treated with VPA at indicated concentrations **(A)** or VE465 at 1 µM, or the combination of VPA at 2 mM and VE 465 at 1 μM **(B)** for 72 h within the same experiment. Cell pellets were harvested and suspended in 1× Annexin V binding buffer at a concentration of 1 × 10^6^ cells/ml. Following incubation of 100 µl of the mixed solution containing 5 µl of FITC Annexin V and 5 µl PI for 15 min at room temperature in dark, 400 µl of 1× binding buffer was added to each tube. Specimens were analyzed using a Gallios Cell Analyzer (Beckman Coulter Gallios™). Data represent means of three experiments with error bars to represent SEM. The combination of VE 465 and VPA increased apoptosis significantly in 2008/C13 cells, compared to the controls or single drug (**p < 0.001, by ANOVA).

**Figure S2:**
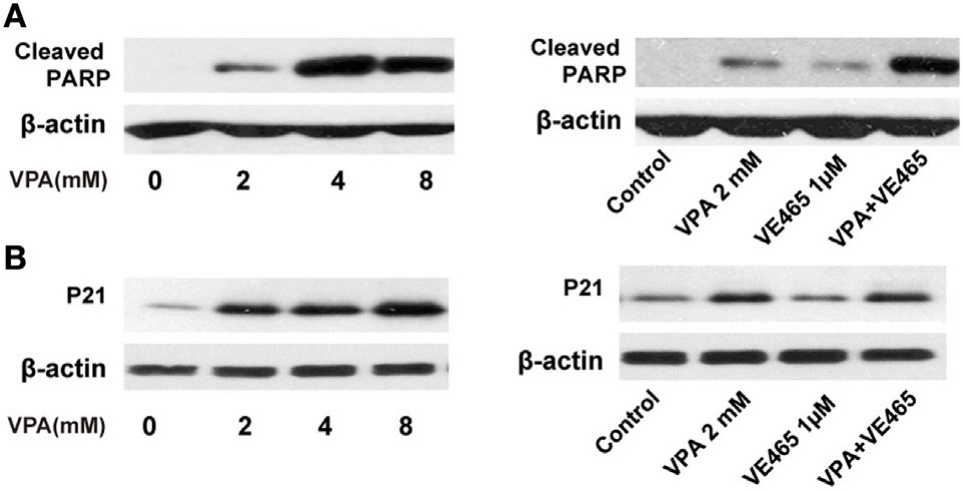
(relates to Figure 6). Western blot analysis of cleaved PARP **(A)** and P21 expression **(B)** by 72 h treatment with VPA alone or in combination with VE465 in 2008/C13 cells. 2008/C13 cells (3 × 10^5^ per well) were plated on 6-well plates overnight. The cells were treated with VPA at indicated concentrations or VE465 at 1 µM, or the combination with VPA at 2 mM and VE 465 at 1 µM for 72 h within the same experiment. Western blot analysis was carried out as described in the original paper. The experiments were repeated two times. Western blot analysis confirmed that VPA induced p21 expression in 2008/C13 cells, compared to the controls. Combination of VPA and VE680 (VE465) induced more cleaved PARP, compared to the control, or single drug.

## Conflict of Interest Statement

The authors declare that the research was conducted in the absence of any commercial or financial relationships that could be construed as a potential conflict of interest.

