# Supplementary material for: Enhanced Cytotoxic Effects of Combined Valproic Acid and the Aurora Kinase Inhibitor VE465 on Gynecologic Cancer Cells
Source: Front Oncol. 2013 Mar 20;3:58. doi: 10.3389/fonc.2013.00058 (PMC3602963; doi:10.3389/fonc.2013.00058)
Supplement: Supplementary file 1 [file Image_1.PDF]

Supplementary documents

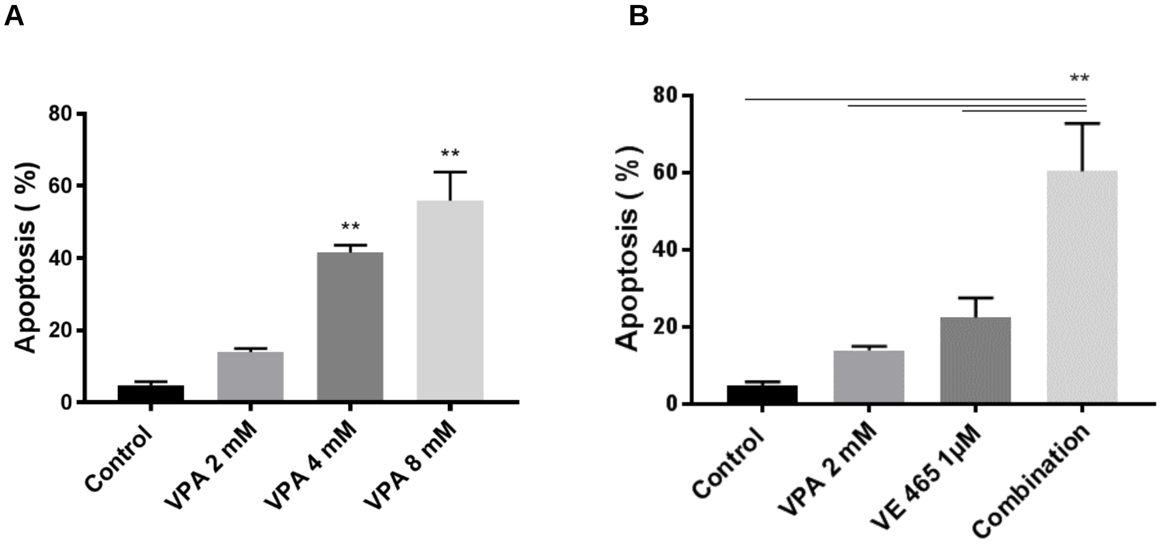

Supplementary Figure 1 (relates to Figure 5). Annexin V analysis of apoptosis induced by VPA alone or in combination with VE 680 (VE465) for 72 h in 2008/C13 cells. Apoptotic cells were evaluated using an Annexin V apoptosis detection kit (BD Biosciences) according to the manufacturer's protocol. Briefly, 2008/C13 cells ($3x10^5$ per well) were plated on 6-well plates overnight. The cells were treated with VPA at indicated concentrations (A) or VE465 at 1 µM, or the combination of VPA at 2 mM and VE 465 at 1 µM(B) for 72 hours within the same experiment. Cell pellets were harvested and suspended in 1X Annexin V binding buffer at a concentration of 1×10^6 cells/ml. Following incubation of 100 µl of the mixed solution containing 5 µl of FITC Annexin V and 5 µl PI for 15 min at room temperature in dark, 400 µl of 1X binding buffer was added to each tube. Specimens were analyzed using a Gallios Cell Analyzer (Beckman Coulter Gallios™). Data represent means of three experiments with error bars to represent SEM. The combination of VE 465 and VPA increased apoptosis significantly in 2008/C13 cells, compared to the controls or single drug (**$p<0.001$, by ANOVA).
