# Supplementary material for: Enhanced Cytotoxic Effects of Combined Valproic Acid and the Aurora Kinase Inhibitor VE465 on Gynecologic Cancer Cells
Source: Front Oncol. 2013 Mar 20;3:58. doi: 10.3389/fonc.2013.00058 (PMC3602963; doi:10.3389/fonc.2013.00058)
Supplement: Supplementary file 2 [file Image_2.PDF]

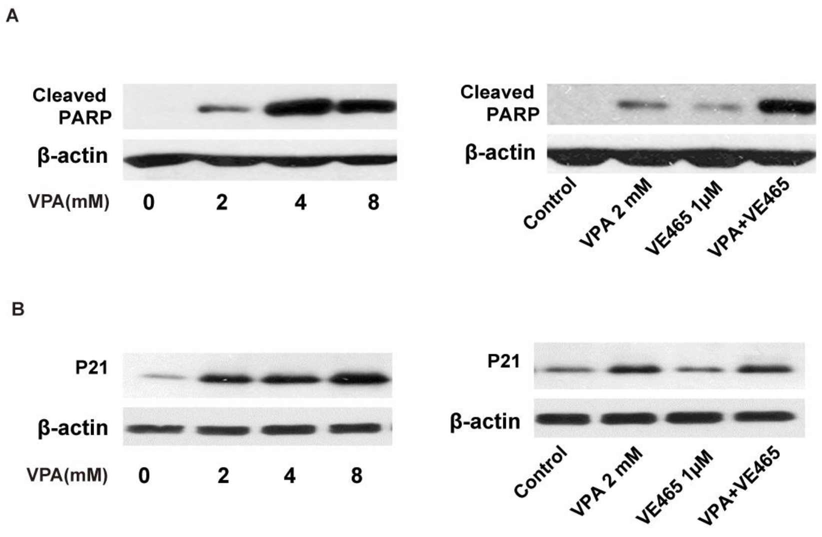

Supplementary Figure 2 (relates to Figure 6). Western blot analysis of cleaved PARP (A) and P21 expression (B) by 72h treatment with VPA alone or in combination with VE465 in 2008/C13 cells. 2008/C13 cells ($3x10^5$ per well) were plated on 6-well plates overnight. The cells were treated with VPA at indicated concentrations or VE465 at 1 µM, or the combination with VPA at 2 mM and VE 465 at 1 µM for 72 hours within the same experiment. Western blot analysis was carried out as described in the original paper. The experiments were repeated two times. Western blot analysis confirmed that VPA induced p21 expression in 2008/C13 cells, compared to the controls. Combination of VPA and VE680 (VE465) induced more cleaved PARP, compared to the control, or single drug.
